# Investigation of the Properties of High-Viscosity Modified Asphalt Binder under Hygrothermal Environments

**DOI:** 10.3390/ma17122869

**Published:** 2024-06-12

**Authors:** Mingliang Xing, Guimin Li, Xiaowei Zhou, Huan Liu, Zhulin Cao, Zuzhong Li, Huaxin Chen

**Affiliations:** 1 School of Materials Science and Engineering, Chang’an University, Xi’an 710064, China; mlxing@chd.edu.cn (M.X.); hxchen@chd.edu.cn (H.C.); 2 Anhui Road & Bridge Engineer Co., Ltd., Pavement Branch, Hefei 230031, China

**Keywords:** high-viscosity modified asphalt binder, polymer modifier, hygrothermal cycling aging test, thermal stability, rheological property

## Abstract

High-viscosity modified asphalt binder (HVMA) is used widely as a polymer-modified binder in porous asphalt pavement because it can improve the cohesiveness of the asphalt mixture. However, because of the high voidage in the mixture, HVMA is vulnerable to aging induced by temperature, oxygen, water, sunlight, and other climatic conditions, which degrades the performance of pavement. The properties of asphalt binder are affected adversely by the effects of hygrothermal environments in megathermal and rainy areas. Therefore, it is essential to study the aging characteristics of HVMA under the influence of hygrothermal environments to promote its application as a high-viscosity modifier. A hygrothermal cycle aging test (HCAT) was designed to simulate the aging of HVMA when rainwater was kept inside of the pavement after rainfall in megathermal areas. One kind of base bitumen and three kinds of HVMA (referred to as SBS, A, and B, respectively) were selected in this study. Short-term aging tests, hygrothermal cycling aging tests, and long-term aging tests were performed on the base bitumen and three kinds of modified asphalt binder. Fourier-transform infrared spectroscopy (FTIR), thermo-gravimetric analysis (TGA), and dynamic shear rheological (DSR) tests were used to evaluate the properties of the binders on the micro and macro scales. By comparing the index variations of the four binders before and after aging, the effects of the hygrothermal environment on the properties of HVMA were studied. It was found that the effects of the hygrothermal environment expedited the decomposition of the polymer and the formation of carbonyl groups compared with the TFOT and PAV test, which TGA confirmed further. Moreover, the thermal stability of the samples was improved after HCAT. In addition, the master curves of the complex modulus showed that hygrothermal cycles made the high-temperature rutting resistance of asphalt binder increase significantly. All of the results above verified that the effect of hygrothermal cycling could accelerate the aging of HVMA and shorten its service life.

## 1. Introduction

Porous asphalt pavement can improve the skid resistance of the surface layer due to its high voidage and deep surface texture, especially on rainy days [1,2]. High-viscosity asphalt binder is needed to ensure the performance of the mixture. High-viscosity asphalt binder, as an important part of porous asphalt pavement, plays a significant role in asphalt mixtures, the properties of which determine the performance of porous asphalt pavement [3,4]. In hygrothermal environments, asphalt binder viscosity decreases and fluidity increases, and the deformation resistance of porous asphalt pavement and the adhesion between asphalt binder and aggregate can be easily decreased [5]. High-viscosity asphalt binder is also prone to aging because of the effects of exoteric factors, which causes the degradation of performance and pavement damage.

The performance of porous asphalt pavement is determined by the properties of the materials and environmental factors. In recent years, several profound studies have been conducted on the influence of environmental factors on the properties of HVMA. Ma et al. [6] studied the effect and mechanism of moisture aging on asphalt binder using macroscopic and microscopic tests. The results showed that moisture had a significant accelerating effect on binder aging, with viscosity increasing 19.6% for base asphalt and 11.9% for SBS-modified asphalt binder. And the results of FTIR showed that the carbonyl index increased by 13.5% and 14.5%, respectively, after moisture PAV aging compared with dry PAV aging. Geng et al. [7] studied the aging characteristics of SBS modifier and SBS-modified asphalt binder under hot and humid conditions, and found that under wet conditions, the carbonyl indices of BA and PMA increased by 30% and 20%, respectively, after 20 h of PAV aging relative to the carbonyl indices obtained under dry conditions. Hence, under the actions of heat and oxygen, the presence of water accelerated the aging of asphalt binder and modifier. Qian et al. [8] analyzed the effect of different water environments combined with ultraviolet radiation on asphalt binder aging and found that the carbonyl index of UV-water increased by 6.7% compared with that of UV radiation with a 395 nm wavelength, which showed the aging was accelerated in the water environment under UV. At the same time, the influence of UV wavelength on asphalt binder aging was different.

The purpose of this research is to study HVMA aging under hygrothermal environments and provide theoretical support for the performance deterioration of drainage asphalt pavement in megathermal and rainy areas. In this study, three kinds of HVMA binders (referred to as SBS, A, and B) were prepared using SK-70 as the base bitumen. Short-term aging, hygrothermal cycling aging, and long-term aging tests were carried out. On this basis, the trend in the chemical structure evolution of the four kinds of asphalt binder after aging was determined by FTIR, and the thermal stability of the samples was assessed by TGA. The DSR test was adopted to analyze the rheological properties of samples before and after aging. Finally, the correlation between structure evolution and performance evolution of high-viscosity asphalt binder was determined.

## 2. Materials and Methods

### 2.1. Materials

#### 2.1.1. Bitumen

The base bitumen used in this study was SK-70 (denoted as SK), which was produced in Korea. The physical properties of SK-70 were tested according to the Chinese standard specification JTG F40–2004. Table 1 shows its basic physical properties.

**Figure 1:**
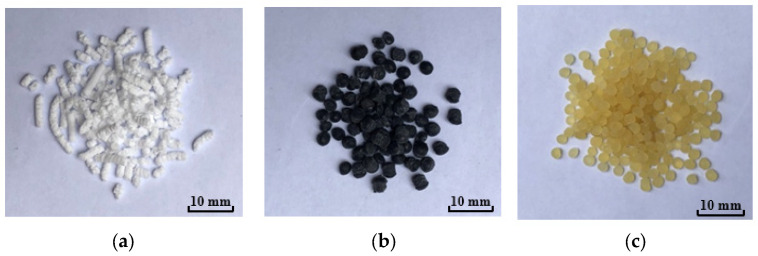
Different modifiers: (**a**) SBS modifier; (**b**) A modifier; and (**c**) B modifier.

To study the influence of different high-viscosity modifiers on asphalt binder performance, two kinds of polymer modifiers (A and B) were selected as experimental groups, with SBS modifier selected as a control group. The SBS modifier consisted of white, long cylindrical particles (Figure 1a), A modifier was black spheres (Figure 1b), and B modifier was light-yellow spheres (Figure 1c).

#### 2.1.2. Preparation of Modified Asphalt Binder

**Figure 2:**
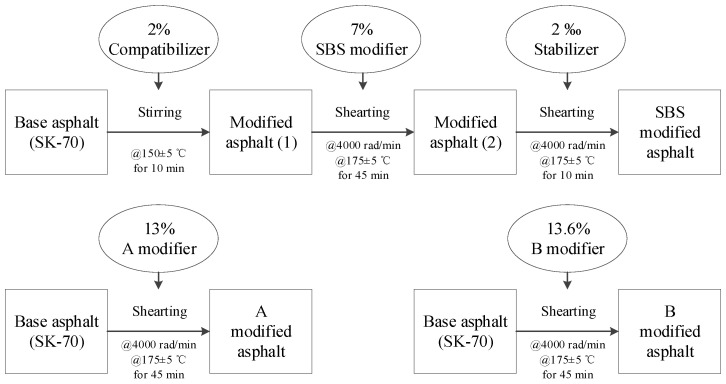
Flow chart of modified asphalt binder preparation.

The preparation process of HVMA is shown in Figure 2, while Table 2 shows the technical indices of SBS-modified asphalt binder (SBS), A-modified asphalt binder (A), and B-modified asphalt binder (B).

### 2.2. Aging Test

#### 2.2.1. Heat Aging Test

According to the requirements of ASTM D1754 (ASTM D1754, 2009) and ASTM D6521 (ASTM D6521, 2013), standard TFOT and PAV tests were carried out for SK, SBS, A, and B, respectively. The codes of the samples after the TFOT or PAV process were followed by the corresponding “-T” and “-P”, respectively (e.g., SK-T and SK-P).

#### 2.2.2. Hygrothermal Cycling Aging Test

In hot areas, pavement retains much rainwater in the structural layers and is subjected to thermo-oxidation after rainfall. The hygrothermal aging test (HCAT) was designed in this study to simulate the aging behavior of HVMA under a hygrothermal environment. Firstly, the asphalt binders were aged by short-term aging (TFOT). Then, each sample was poured into a standard 2 L beaker until its thickness was 2 mm. The beakers were placed in an oven at 150 °C to ensure that the asphalt binder could be laid, and the samples were removed from the oven after their thickness was uniform.

**Figure 3:**
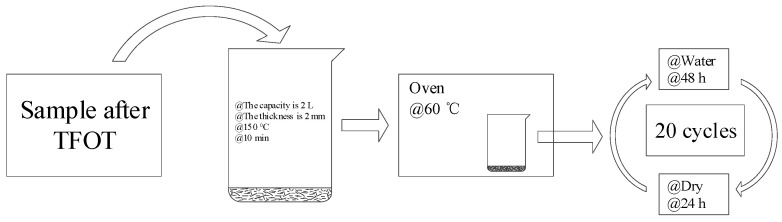
Flow chart of HCAT.

The HCAT method was based on climate and environmental characteristics, and the circulation mechanism of “water” was adopted in a heated environment. The specific methods used were as follows. Abundant water was added to the beaker containing the asphalt binder sample to prevent the water from evaporating completely, after which it was placed in the oven, which was regarded as a moisture-aging process. The temperature in the oven was maintained at 60 °C for 48 h. After 48 h of moisture-aging, the residual water was poured from the beaker and the samples were kept in the oven at 60 °C for 24 h, which was the thermal aging process. This was one cycle. HCAT lasted for 20 cycles, in which one cycle consisted of one moisture aging process and one thermal aging process. The flow chart is shown in Figure 3. The codes of the samples after HCAT were followed by the corresponding “-W” (e.g., SK-W).

### 2.3. FTIR

A Bruker EQUINOX-55 infrared spectrometer (Bruker, Saarbrucken, Germany) was used in this study. At least three replicates were measured for each bitumen sample. The wavenumber range was from 4000 cm^−1^ to 600 cm^−1^. Based on infrared spectroscopy, the characteristic absorption peaks of various functional groups in the asphalts could be analyzed quantitatively. After the spectra were collected, the infrared data were processed in OMNIC (8.0, Thermo Fisher Scientific, Waltham, MA, USA). By analyzing the changes of the functional groups in the specimens, the effects of HCAT on the chemical structures of the asphalt binders could be well elucidated. After the spectra were collected, the infrared data were processed in OMNIC.

### 2.4. Thermal Analysis

The thermal stability of asphalt binder can be analyzed by TGA (TA Instruments, New Castle, DE, USA), as was performed in this work. The SDT650 thermo-gravimetric analyzer was used in a furnace at a constant heating rate of 20 °C/min in a nitrogen atmosphere. The sample of 5 mg was heated from 25 °C to 800 °C with a nitrogen flow rate of 100 mL/min. The relationship between the thermogravimetric properties and temperature was used to determine the thermal stability of the samples.

### 2.5. Rheological Study

#### 2.5.1. Temperature Scanning in DSR

A DHR-1 dynamic shear rheometer (TA Instruments, New Castle, DE, USA) was used to scan the temperature of the asphalt binders after the non-aging, TFOT, HCAT, and PAV tests. According to AASHTO TP5-93, the strain control mode was adopted. The samples were subjected to sinusoidal shear loading at a shear rate of 10 rad/s. The temperature scan range was 46–82 °C and the interval was 6 °C.

#### 2.5.2. Frequency Scanning in DSR

The frequency scanning test was also carried out with the same rheometer. The frequency scanning test is the primary method used to study asphalt binder viscoelasticity, and it was measured in this study at 46 °C, 52 °C, 58 °C, 64 °C, 70 °C, 76 °C, and 82 °C. The loading frequency range was 0.1–100 rad/s, and a dynamic shear load with a low strain level of 1.5% was applied to the asphalt binders. The combination of temperature and frequency ensured sufficient overlap in the material response. Therefore, the master curve could be obtained by data fitting.

The master curve can be established by a mathematical model and a mechanical model [9]. The Christensen–Anderson–Marasteanu model [10,11] was selected in this work.

The shift factor, αT, is calculated using the Williams–Landel–Ferry (WLF) equation [12], as shown in Formula (1):

(1) $$log\alpha_{T}\left(T\right)=-\frac{C_{1}(T-T_{0})}{C_{2}+(T-T_{0})}$$

where *C*_1_ and *C*_2_ are model parameters, and *T*_0_ is the reference temperature.

#### 2.5.3. MSCR

The multiple Stress Creep and Recovery (MSCR) testing method covers the determination of percent recovery and non-recoverable creep compliance of asphalt binder. The MSCR test is conducted using a dynamic shear rheometer (DSR) at a specified temperature [AASHTO T350-14 (2018) Standard Method of Test for Multiple Stress Creep Recovery (MSCR) Test of Asphalt Binder Using a Dynamic Shear Rheometer (DSR) [S]. Washington, DC, USA: AASHTO, 2018]. The MSCR test can well demonstrate the deformation performance of polymer-modified asphalt binder [13]. It was also performed with DHR-1. The tests comprised 10 consecutive cycles, and each cycle consisted of a 1 s creep period, followed by a 9 s recovery period. The samples used for the MSCR tests were 25 mm diameter and 1 mm thickness cylinders. It was also performed with DHR-1. According to ASTM D7405, two constant stresses (0.1 kPa and 3.2 kPa) were used in the creep and recovery tests at 58 °C, 64 °C, 70 °C, 76 °C, and 82 °C, respectively. The recovery percentage (R) and irrecoverable creep compliance (J_nr_) at 0.1 kPa and 3.2 kPa were obtained by calculation. They were recorded as R_0.1_, J_nr 0.1_, R_3.2_, and J_nr 3.2_, respectively. The samples used for the MSCRs test were 25 mm diameter and 1 mm thickness cylinders.

## 3. Results and Discussion

### 3.1. FTIR Analysis

**Figure 4:**
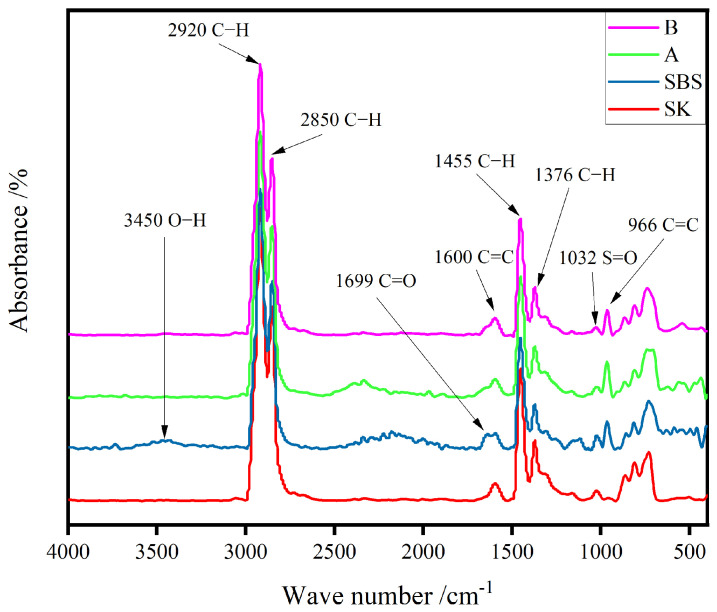
FTIR spectra of unaged asphalt binders.

In this work, carbonyl and butadiene functional groups were mainly analyzed. The former was used to characterize the oxidation degree of the asphalt binders, and the latter was used to characterize the degradation of the polymer [14,15]. Figure 4 shows the infrared spectrum contrast map of four kinds of unaged asphalt binder. The functional group information is marked only in Figure 4. The main functional group information was determined and is listed in Table 3.

**Figure 5:**
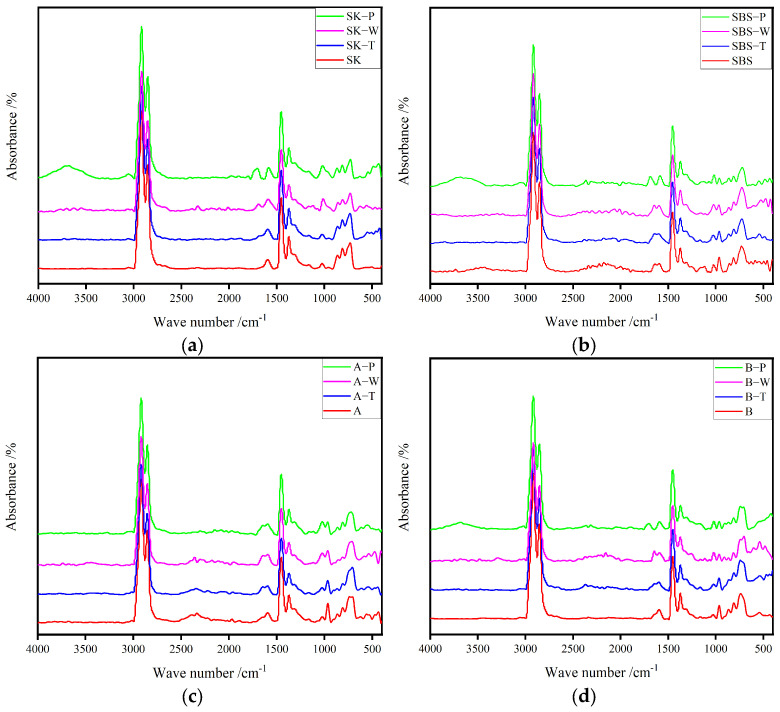
FTIR spectra of different asphalt binder samples before and after aging: (**a**) SK; (**b**) SBS; (**c**) A; and (**d**) B.

Figure 5 shows the FTIR curves of the four kinds of asphalt binder before and after aging. The aging degree was characterized by the changes in the spectrum, which mainly included the spectral changes of two characteristic bands: the carbonyl functional group (center near 1699 cm^−1^) and the butadiene functional group (center approximately 966 cm^−1^) [16]. Affected by heat, pressure, and moisture, the aging of modified asphalt binders sped up, leading to a series of oxidizing reaction from asphalt components [17]. It could be seen that the peak of the carbonyl group rose obviously as the aging degree of the asphalt binders deepened. Moreover, the peak of the butadiene functional group tended to decrease, which was an indicator of degradation level for the polymer modifiers [18].

A semi-quantitative analysis of these two functional groups helped to determine the aging behavior of these four samples [19]. Several structural indices were calculated using the following equations:

(2) $$I_{C=O}=\frac{A_{1699}}{\sum A}$$

(3) $$I_{C=C}=\frac{A_{966}}{\sum A}$$

where A_1699_ is the carbonyl peak area centered at 1699 cm^−1^, and A_966_ is the butadiene peak area centered at 966 cm^−1^. Σ A is the total area of the peak with wavenumbers between 2800 cm^−1^ and 3000 cm^−1^. The total area of the peak was unchanged before and after aging.

**Figure 6:**
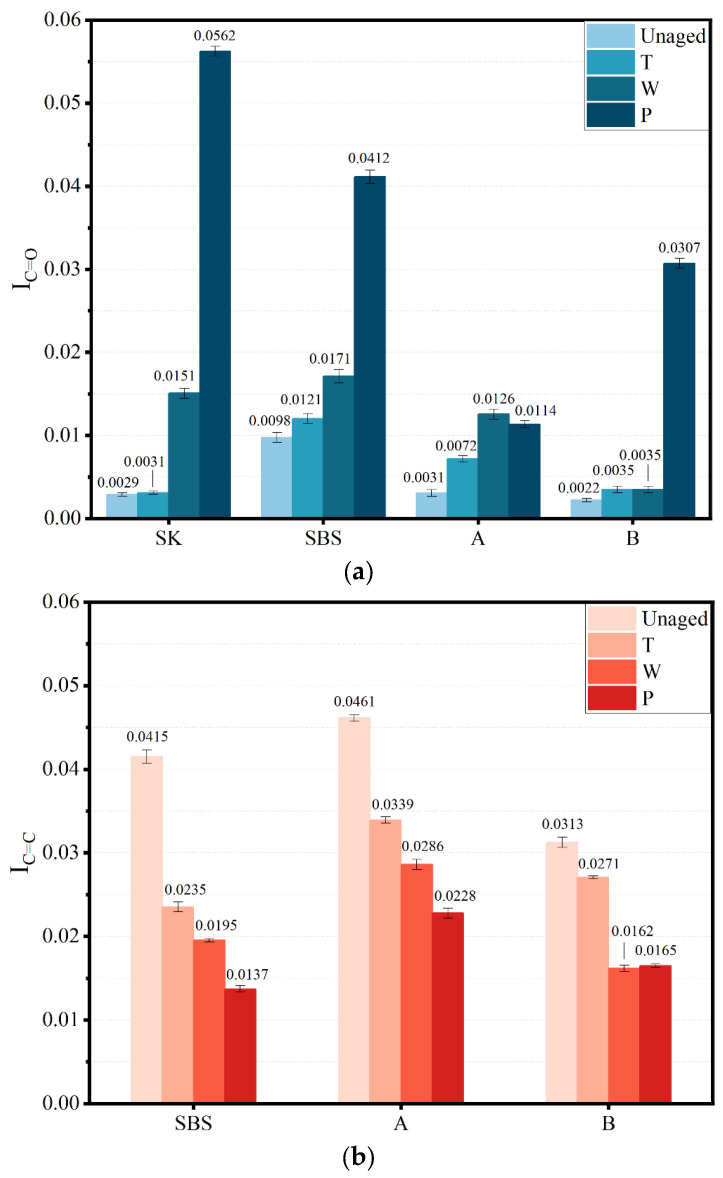
Peak band structure index of different asphalt binder samples: (**a**) carbonyl index; and (**b**) butadiene index.

Figure 6 shows the carbonyl and butadiene indices of the asphalt binders in different aging states, respectively. It could be found in the figure that the carbonyl index of various samples increased, while the butadiene index declined. Generally, these modifiers were composed of some polymers, containing plenty of unsaturated -C=C-. During modification, polymers adsorbed the light components of asphalt and delayed the aging of asphalt. Moreover, another reason was that the unsaturated -C=C- in butadiene was oxidized and formed a carbonyl group [14]. It should be noted that there were no butadiene groups in the original SK. However, the carbonyl index of SK after aging increased significantly, attributing to the rupture of C-C from asphalt components and facilitating the formation of carbonyl group. Besides, due to complexity of asphalt materials, the partial hydroxy goup from asphalt components could be oxidized and increased the content of carbonyl groups.

To research the effect of aging on performance, the change rate of the aging index was calculated according to Equations (4)–(9), as follows:

(4) $$\Delta T,I_{C=O}=\frac{{\left(I_{C=O}\right)}_{TFOT}-{\left(I_{C=O}\right)}_{ORIGIN}}{{\left(I_{C=O}\right)}_{ORIGIN}}\times 100\%$$

(5) $$\Delta H,I_{C=O}=\frac{{\left(I_{C=O}\right)}_{Hygrothermal}-{\left(I_{C=O}\right)}_{ORIGIN}}{{\left(I_{C=O}\right)}_{ORIGIN}}\times 100\%$$

(6) $$\Delta P,I_{C=O}=\frac{{\left(I_{C=O}\right)}_{PAV}-{\left(I_{C=O}\right)}_{ORIGIN}}{{\left(I_{C=O}\right)}_{ORIGIN}}\times 100\%$$

(7) $$\Delta T,I_{C=C}=\frac{{\left(I_{C=C}\right)}_{TFOT}-{\left(I_{C=C}\right)}_{ORIGIN}}{{\left(I_{C=C}\right)}_{ORIGIN}}\times 100\%$$

(8) $$\Delta H,I_{C=C}=\frac{{\left(I_{C=C}\right)}_{Hygrothermal}-{\left(I_{C=C}\right)}_{ORIGIN}}{{\left(I_{C=C}\right)}_{ORIGIN}}\times 100\%$$

(9) $$\Delta P,I_{C=C}=\frac{{\left(I_{C=C}\right)}_{PAV}-{\left(I_{C=C}\right)}_{ORIGIN}}{{\left(I_{C=C}\right)}_{ORIGIN}}\times 100\%$$

The change rates of aging are listed in Table 4. As the aging degree deepened, the values of I_C=O_ increased, while the values of I_C=C_ decreased. It could be seen in the table that the aging indices of the four kinds of asphalt binder differed significantly. In HCAT, the effect of oxygen on asphalt binder was weakened due to the inhabitation of water. However, there was still some oxygen in the water, and the aging degree of the samples was more serious than after TFOT. Hygrothermal environment factors could not be ignored. A large number of -C=C- in the butadiene functional group could be oxidized in a hygrothermal environment, which thus produced more polar groups [17]. Obviously, the butadiene aging index of SBS, A, and B asphalt binders decreased by 53%, 38%, and 48%, respectively, after HCAT. The degradation of the polymer in HCAT was close to that in long-term aging. For the SK, SBS, A, and B samples, the carbonyl index increased by 416%, 76%, 308%, and 741%, respectively, after HCAT. The increase in the carbonyl groups was caused by the comprehensive influence of the oxidation of asphalt binder and the decomposition of polymers. It could be concluded that the oxidation in asphalt binder was accelerated due to the presence of water.

### 3.2. Thermal Analysis

**Figure 7:**
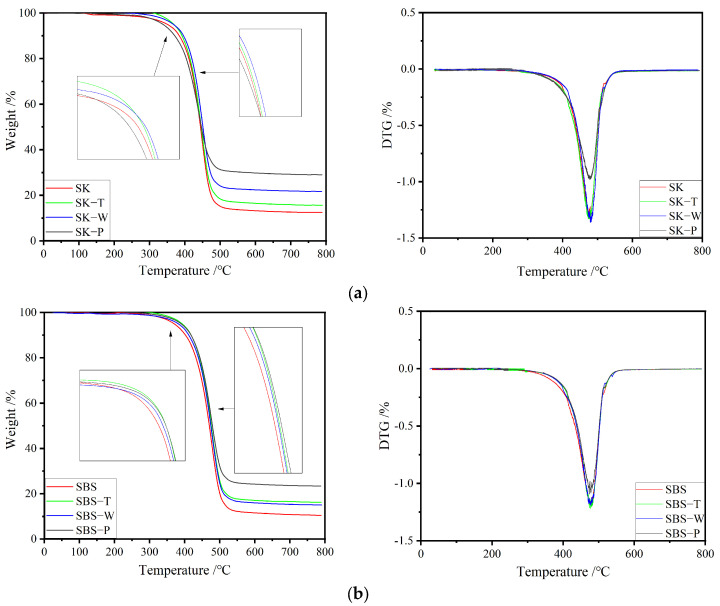

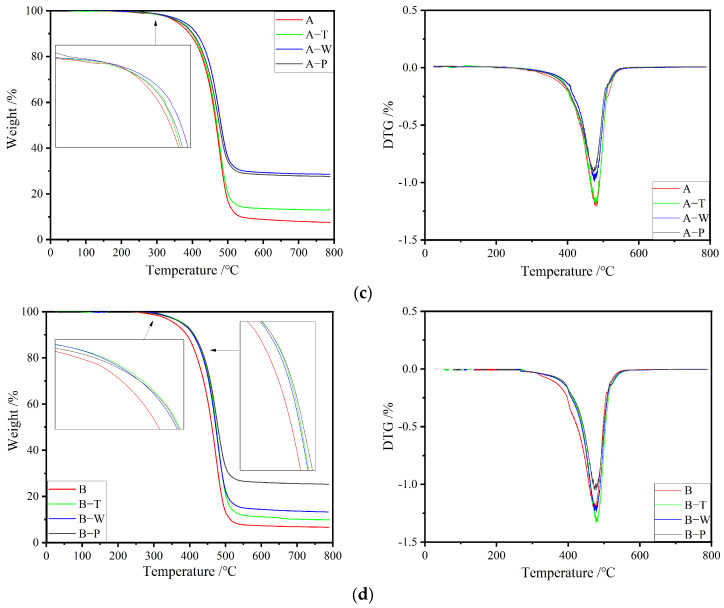
TG curves and DTG curves of different pre- and post-aging asphalt binder samples: (**a**) SK; (**b**) SBS; (**c**) A; and (**d**) B.

Figure 7 shows the TG and DTG curves of different samples, and it was obvious that all curves demonstrated a single mass loss process.

The curves of all samples displayed three main weight loss stages. The initial stage corresponding to 0–200 °C approximated a horizontal line. In this stage, the mass of asphalt binder scarcely varied, and there was almost no chemical reaction. In the second stage (i.e., the corresponding curve between 200 and 550 °C), light components volatilized, while heat decomposed the asphaltene and asphalt modifiers and caused the asphalt binder to lose a total mass of over 70% [14]. The last stage occurred from 550 to 800 °C with a small mass loss that was attributable largely to the further volatilization and carbonization of the decayed residues of hard asphalt binder components and modifiers [20,21]. There were imprecise multiple peaks at the top of the DTG curve. As the mass loss in this stage was realized through different steps, the characteristics of each component of asphalt binder would be sensitively reflected in the peak value of DTG [22].

The temperature at which mass loss began increased significantly after aging, which demonstrated clearly that the thermal stability of asphalt binder had been improved. This was because of the increased composition of hard asphalt, such as asphaltene or resin [23]. A comparison of the TG curves of the original sample and the asphalt binder samples after TFOT, HCAT, and PAV tests showed that the residual content at 780 °C had a significant positive correlation with the aging degree [24].

Table 5 shows the TG/DTG results of the samples. After HCAT, the temperature increased slightly when the mass loss reached 10% (T_10 wt.%_), the mass loss decreased at 200–550 °C, and the content of residues of HVMA at 780 °C was higher. This showed that the effects of the hygrothermal environment promoted the aging of HVMA, and the aging degree of different kinds of asphalt binder differed. This result was consistent with the generation of C=O and the weakening of C=C shown in the FTIR spectra in the previous section. For each modified binder, the influence of the increase in asphaltene content was predominant in the thermal stability of the binder during the short-term aging process (TFOT). Compared with that of the binders after TFOT, the maximum residue content at 780 °C increased significantly after HCAT and PAV tests. There were two main reasons for these results. The first was the loss of volatile components in asphalt binder during aging [25], while the second was that the thermal stability of HVMA was enhanced after the HCAT or PAV tests. This indicated that the further decomposition of the polymer in the process of HCAT and PAV tests had no obvious effect on the dominant role in the increase in asphaltene [26].

### 3.3. Rheological Analysis

#### 3.3.1. DSR Analysis

##### Rutting Factor Analysis

**Figure 8:**
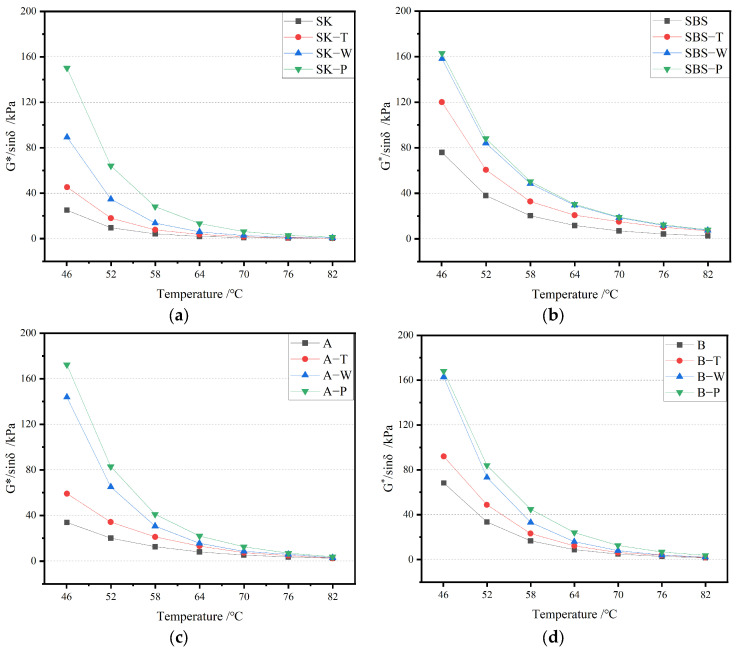
Relationship between rutting factor and temperature of asphalt binders after different aging processes: (**a**) SK; (**b**) SBS; (**c**) A; and (**d**) B.

Based on SHRP specifications, the rutting factor (G*/sin δ) was selected as the parameter that represented the anti-rutting performance. The greater the value of G*/sin δ, the greater the resistance to rutting of the asphalt binder [26]. G*/sin δ of all samples could be measured through the test. The relationship between the rutting factor and temperature of asphalt binder after different aging methods is shown in Figure 8.

As the temperature rose, the G*/sin δ value of asphalt binder showed a downward trend. This showed that the resistance to rutting performance of the asphalt binder declined with the rise in temperature. However, when it reached a certain temperature, the G*/sin δ value decreased slowly and tended to be stable, attributed to the movement of molecules speeding up with the increase in temperature. The external force required to produce the same deformation of asphalt binder decreased, which was manifested as a decline in the rutting factor [27]. It can also be seen in the figure that the G*/sin δ values of the four asphalt binders increased after aging at the same temperature, inferring that the anti-rutting performance of asphalt binder increased after aging.

##### Analysis of Complex Modulus Master Curve

**Figure 9:**
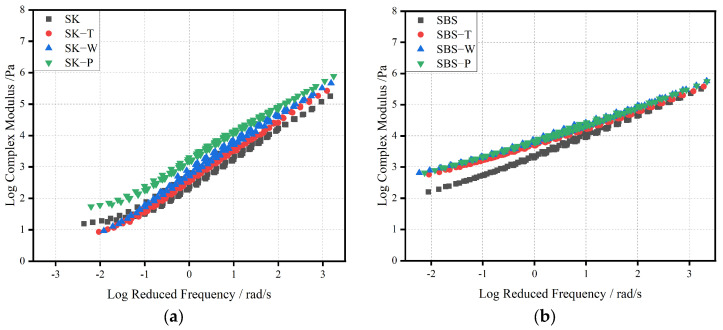

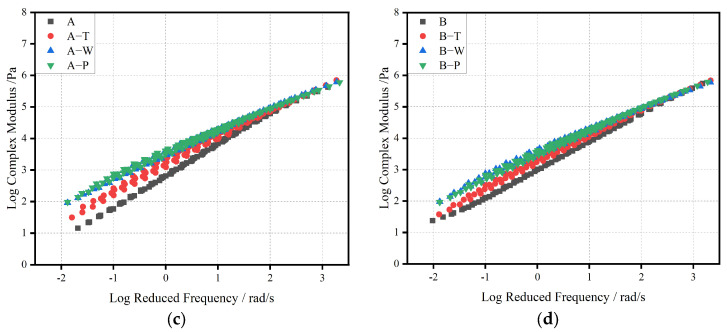
Master curves of asphalt binders after different aging processes: (**a**) SK; (**b**) SBS; (**c**) A; and (**d**) B.

The master curve is one of the most frequently used methods for studying the linear rheological behavior of asphalt binder [28]. According to the principle of time–temperature equivalence, the master curve was synthesized by horizontal displacement superposition. The 64 °C broadband master curves of frequency versus complex modulus were obtained, as shown in Figure 9a–d.

It can be seen that regardless of the aging method, the complex modulus of asphalt binder increased with the increase in frequency. With the increase in test frequency, the influence of aging on the complex modulus weakened gradually until all of the master curves met. This was because high frequency (low temperature) limited the movement of asphalt molecules [29]. The same trend as in the results above proved further that hygrothermal cycling aging had an obvious effect on the rheological properties of the three modified asphalt binders. The complex modulus of HVMA after HCAT was similar to that of the PAV test, while the PAV test had a greater influence on the base bitumen.

It could be seen from Figure 9a that the master curves of SK moved upward after aging. Moreover, the curves in the low-frequency (high-temperature) region showed the greatest change. This showed that aging affected the high-temperature properties of SK. In the low-frequency region, the master curves of samples after TFOT and WACT were quite similar, and they were lower than the curve of the sample after the PAV test. Thus, long-term aging had the most serious effect on the complex modulus in the low-frequency region. This conclusion was the same as in the previous section. It could be seen from Figure 9b–d that the master curves of HVMA moved upward after aging. Because their aging resistance was greater, the change range of the master curve after aging was less than that of SK. For sample A, the modulus master curves corresponding to the different aging methods were different in the low-frequency range. As the frequency increased, the discrepancy between hygrothermal cycling aging and long-term aging decreased. The severity of aging was ranked as P > H > T. The trend of the curves of sample B was similar to that of sample A.

Therefore, it could be seen from the master curves that the high-temperature rutting resistance of asphalt binder was significantly promoted at a macro level and was affected by the effects of the hygrothermal environment.

#### 3.3.2. MSCR Analysis

**Figure 10:**
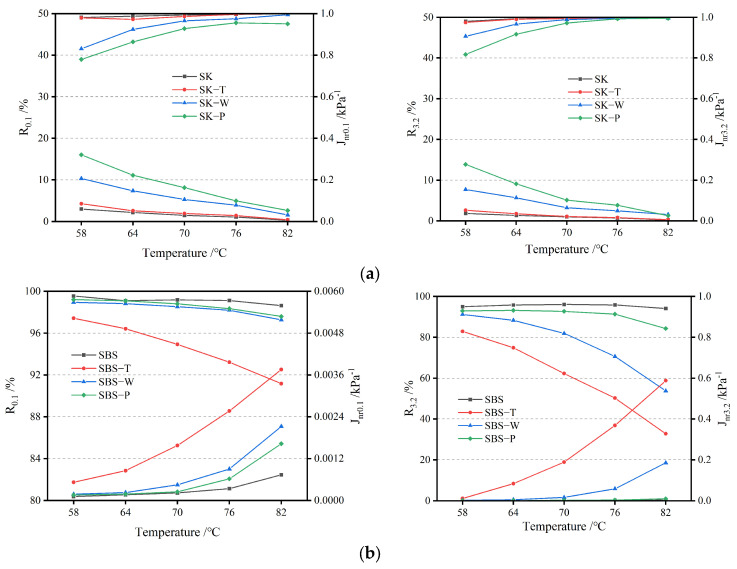

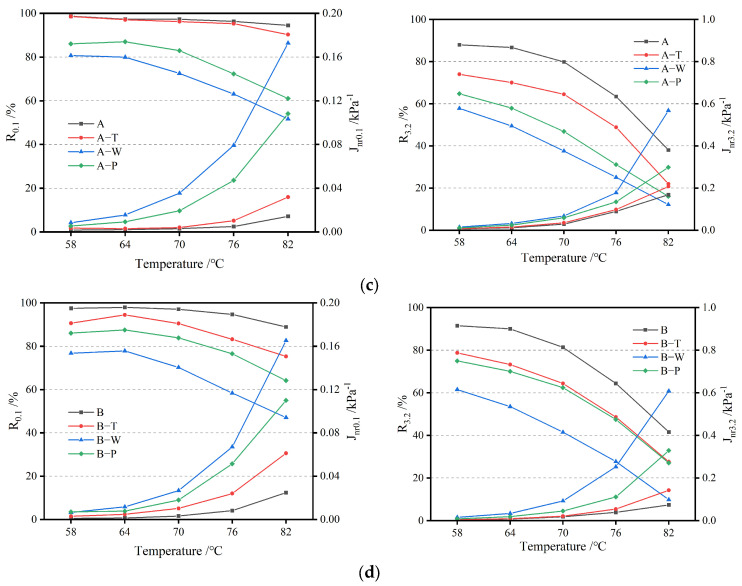
MSCR results of asphalt binders at all test temperatures: (**a**) SK; (**b**) SBS; (**c**) A; and (**d**) B.

The MSCR results of the asphalt binders are presented in Figure 10. As for high-viscosity modified asphalt, the upper part in each chart was the strain recovery rate (R), while the lower part in each chart was the creep compliance (J).

The behavior of the base bitumen was quite simple, and its elasticity increased with aging. As a result, the R value increased and the J_nr_ value decreased. Under the same aging conditions, the R value of SK exhibited a downward trend with the rise in temperature, and the variation of the J_nr_ value was the opposite. This showed that the elasticity of asphalt binder decreased and pure asphalt binder was not sensitive to the magnitude of the shear force.

Figure 10 shows that the strain recovery rate (R) of all asphalts decreased with the rise in temperature, while the creep compliance (J) of all asphalts increased with the rise in temperature. Modified asphalt consists of two phases, including the asphalt phase and the modifier phase. On the whole, for bitumen, as the temperature rose, the viscous components in the asphalt phase increased. During short-term aging, asphalt aging and polymer degradation were not obvious. As for high-viscosity modified asphalt with a high dosage of modifiers, the viscosity of the asphalt phase decreased at higher temperatures, leading to the modifier phase being more easily deformed [30]. Consequently, the interaction between the asphalt and modifier phases weakened, resulting in a reduction in the overall elastic recovery performance of the modified asphalt.

It was also found that the R of base bitumen tended to decrease with the rise in temperature, and the R of the three kinds of HVMA tended to increase first and then decrease, which demonstrated that the matrix asphalt binder was almost completely viscous under stress. The delayed elastic recovery of HVMA weakened gradually when it reached a certain temperature [31].

The SBS, A, and B samples had different trends from the base bitumen. After TFOT, the SBS sample showed the minimum elastic response. At the same time, it had the largest J_nr_ and the minimal R at the two shear forces and all test temperatures. This was consistent with the trend in the previous DSR analysis.

As for HCAT and PAV, the degradation of the polymer was slowed, as was observed in the FTIR test, and the dominant oxidation made the binder harder and more elastic. Further, it was observed that a higher test temperature (82 °C) and higher strain level (3.2 kPa) made the parameter evolution more obvious [32]. It should be pointed out that HCAT had the most serious effect on the viscoelasticity of samples A and B. The reason was that the oxidation of asphalt binder was dominant in the aging process under the hygrothermal environment. However, the degradation of the polymer in A and B was slow during TFOT, while the degradation was aggravated during HCAT.

### 3.4. Correlation Analysis

This section analyzed the correlation between the functional group indices and rheological properties. The functional group indices (I_C=O_ and I_C=C_) of SK, SBS, A, and B before and after aging were fitted linearly with the rheological energy parameters (rutting factor at 64 °C).

The Pearson correlation coefficient (PCC) is used to measure the relationship between two variables [33] (Shao, L et al., 2021). For two variables, x and y, the expression of the PCC is presented in the following Equation (10):

(10) $$R=\frac{\sum \left(X_{i}-\bar{X}\right)\left(Y_{i}-\bar{Y}\right)}{{\left[\sum {\left(X_{i}-\bar{X}\right)}^{2}\sum {\left(Y_{i}-\bar{Y}\right)}^{2}\right]}^{1/2}}$$

where *x* is the functional group index, *y* is the rutting factor, and $\bar{X}$ and $\bar{Y}$ are the mean values of the test results, respectively. The value of *R* ranges from −1 to 1.

**Figure 11:**
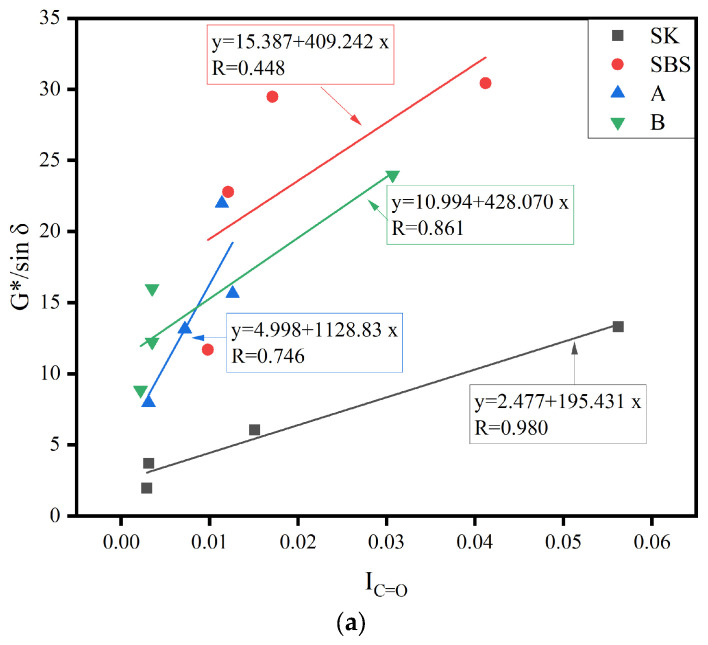

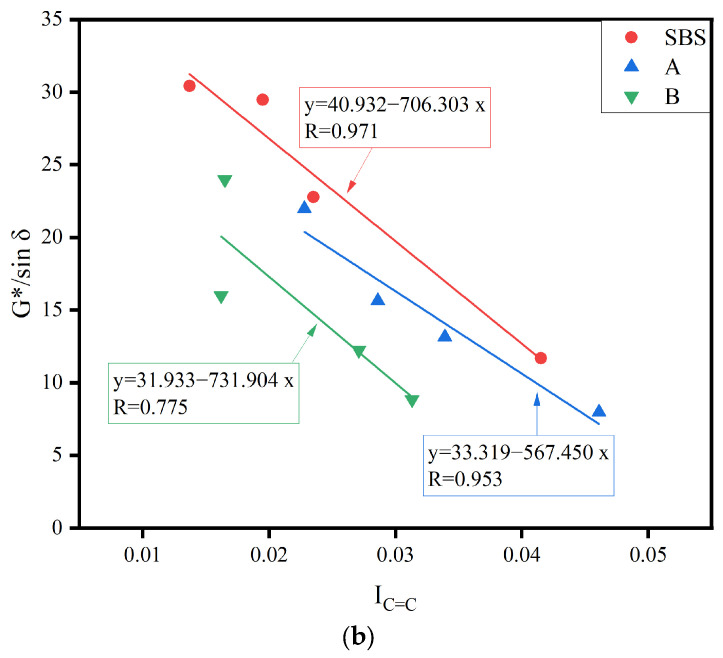
The fitting curves of functional group indices and rheological property parameters: (**a**) I_C=O_; and (**b**) I_C=C_.

As shown in Figure 11, at the 0.05 significance level, the slope of the fitting curve was positive [34]. This showed that C=O had a positive effect on the high-temperature rheological properties of asphalt binder. As we all know, |*R*| ≥ 0.7 when variables are highly correlated. When 0.7 > |*R*| ≥ 0.4, variables are moderately correlated. When |*R*| < 0.4, the correlation is weaker. The *R* values of SK, SBS, A, and B were 0.98, 0.45, 0.75, and 0.86, respectively, which indicated that the micro properties the samples were related to the rheological properties. The SBS samples were moderately correlated, and the other three samples were highly correlated. This may have been due to the serious degradation of SBS modifier. The R values of SBS, A, and B were 0.97, 0.95, and 0.78, respectively, and all of the data were highly correlated.

## 4. Conclusions and Future Work

This work presented a study of the aging behavior and mechanism of four kinds of asphalt binder (i.e., SK, SBS, A, and B) under hygrothermal environments. FTIR, TGA, DSR, and MSCR tests were conducted to determine the effects of various methods (i.e., TFOT, HCAT, and PAV tests) on the asphalt binders. The following conclusions were made.

(1) In FTIR analysis, after aging, the decrease in the butadiene index indicated polymer degradation, while the increase in the molecular weight of asphalt binder led to an increase in the carbonyl index. The effects of the hygrothermal environment expedited the decomposition of the polymer and the formation of carbonyl groups compared with the TFOT and PAV test.
(2) TGA showed that there was only one single mass loss process in all samples, which mainly occurred between 200 and 550 °C. The process involved primarily the decomposition of the residual polymer and hard asphalt binder components. There was a positive correlation between the residual mass at 780 °C and the aging degree of asphalt binder, which corresponded to the change in the carbonyl group in FTIR. The thermal stability of asphalt binder was improved after HCAT. In the hygrothermal environment, the presence of water accelerated asphalt binder aging.
(3) The results of DSR tests showed that the rutting factor increased obviously after HCAT. This indicated that the rutting factor of asphalt binder was significantly affected by the hygrothermal environment, and its anti-rutting performance was elevated. The master curves also verified this point. The results of MSCR tests showed that the elasticity of base bitumen increased with aging, while high-viscosity modified asphalt exhibited a different elastic response to different aging methods. Under the hygrothermal environment, A and B aged further, and there was an elastic response to different aging methods. Under the hygrothermal environment, A and B aged dramatically.
(4) In general, the hygrothermal environment aggravated the aging of asphalt binder and significantly promoted the thermal oxidative aging reaction. Therefore, the service life of high-viscosity modified asphalt in long-term hot and rainy climate areas would be shortened. It is necessary to further improve the performance of the resistance of this material to hygrothermal factors.

In the manuscript, a hygrothermal aging test was designed to simulate the aging behavior of HVMA in a megathermal climate. In the test, we selected a constant temperature of 60 °C to explore the coupling effect of humidity and temperature on the performance of HVMA, which still has some limitations and needs further study. Several sets of nonlinearly varying temperature or humidity test conditions can be designed to better simulate the actual road conditions. Moreover, the performance of HVMA under the single factor of temperature or humidity can be explored and combined with the coupling effect of the two factors to comprehensively investigate the influence of hygrothermal environments on the performance of HVMA.

This study has undertaken some exploration of the structural characteristics and rheological properties of high-viscosity modified asphalt binder under a hygrothermal environment, while demonstrating the impact of hygrothermal cycles in accelerating the aging of HVMA, consequently shortening its service life. In the future, studies should endeavor to investigate the performance of asphalt mixtures by evaluating their behavior under hygrothermal environments and analyzing the effects of complex factors, including time, humidity, loading conditions, and corrosive media.

## Figures and Tables

**Table 1:** Basic physical properties of SK.

Indices	Test Results	Specification Requirement
Softening point/°C	48.5	≥45
Penetration at 25 °C/0.1 mm	66	60–80
Ductility (15 °C)/cm	141	≥100
Dynamic viscosity at 60 °C/Pa·s	213	≥160

**Table 2:** The basic performance of modified asphalt binders.

Indices	Softening Point /°C	Penetration (25 °C)/0.1 mm	Ductility (5 °C) /cm	Dynamic Viscosity (60 °C)/Pa·s	Rotational Viscosity (135 °C)/Pa·s
SBS	118.0	33	33.3	172,676	2.83
Specification Requirement	≥55	30–60	≥30	≥20,000	≤3
A	81.0	46	58.2	140,987	2.43
B	83.0	48	49.7	184,771	2.95
Specification Requirement	≥70	40–80	≥20	≥20,000	≤3

**Table 3:** Peak positions and details of main functional groups.

Peak Position/cm^−1^	Functional Groups	Details
3450	O-H	Stretching vibration
2920	C-H	Asymmetric stretching vibration
2850	C-H	Symmetric stretching vibration
1699	C=O	Stretching vibration
1600	-C=C-	Vibration of Conjugate Ring
1455	CH_3_&CH_2_	Asymmetric deformation
1376	CH_3_	Symmetrical deformation
1032	S=O	Stretching vibration
966	-C=C-	Bending vibration

**Table 4:** The aging index change rates of asphalt binders.

Indices	∆T, I_C=O_/%	∆H, I_C=O_/%	∆P, I_C=O_/%	∆T, I_C=C_/%	∆H, I_C=C_/%	∆P, I_C=C_/%
SK	7	416	1824	/	/	/
SBS	24	76	322	−43	−53	−67
A	132	308	269	−26	−38	−51
B	57	741	1269	−13	−48	−47

**Table 5:** TG/DTG results for pre- and post-aging samples.

Samples	T_10 wt%_/°C	Mass Loss/%	Residue at 780 °C/wt.%
SK	384.0	84.95	12.46
SK-T	391.6	82.55	15.61
SK-W	395.9	76.82	21.69
SK-P	382.8	69.12	29.23
SBS	402.3	87.53	10.45
SBS-T	418.3	82.31	16.17
SBS-W	411.6	82.67	15.04
SBS-P	416.1	75.21	23.37
A	392.1	90.08	7.53
A-T	399.5	85.72	13.22
A-W	412.3	69.93	28.58
A-P	401.6	71.07	27.62
B	391.6	92.26	6.66
B-T	412.7	88.14	9.86
B-W	409.4	84.95	13.28
B-P	412.0	73.38	25.34

## Data Availability

All the data in the tests of this study have been listed in the paper.
